# The roles of output clock genes in regulating glucose metabolism

**DOI:** 10.1111/jdi.14295

**Published:** 2024-10-03

**Authors:** Akihiko Taguchi, Yasuharu Ohta, Yuko Nagao, Yukio Tanizawa

**Affiliations:** ^1^ Department of Endocrinology, Metabolism, Hematological Science and Therapeutics, Graduate School of Medicine Yamaguchi University Ube Japan; ^2^ Health Science Center Yamaguchi University Yamaguchi Japan; ^3^ Yamaguchi University 1677‐1, Yoshida Yamaguchi Japan

## INTRODUCTION

Circadian rhythm is an endogenous autonomous oscillator of physiological activities resulting in 24 h day/night cycles. This rhythm is regarded as a system regulating organisms allowing them to carry out efficient biological activities during the day–night cycle. In humans, the rhythm is set at 24 h and 11 ± 16 min, which is slightly longer than the day rhythm (24 h). Therefore, when living in a dark room, the waking time is slightly delayed each day^1^.

The invention of electric light revolutionized society, with humans now able to work at night, including shift work, and such disruption of the circadian rhythm reportedly increases insulin resistance, as well as raising the risks of type 2 diabetes and cardiovascular diseases^2^, ^3^.

**Figure 1:**
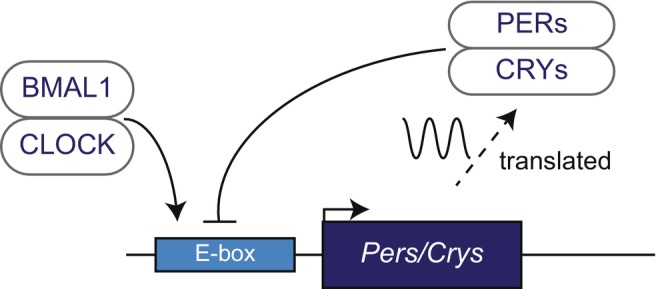
The core transcription–translation feedback loop of the circadian clock. BMAL1/CLOCK binds to the E‐box and enhances the expressions of *Pers* and *Crys*. Translated PERs and CRYS suppress their own gene expressions by inhibiting CLOCK‐BMAL1‐mediated transcription.

In humans, the center of the circadian rhythm is located in the suprachiasmatic nucleus, and the rhythm is generated by a set of genes known as clock genes. The following molecular mechanism generates circadian rhythms: first, the heterodimer of CLOCK‐BMAL1, a set of the core clock gene products, binds to the promoters of the *Per* and *Cry* clock genes, thereby activating both *Per* and *Cry* transcription. The translated PER and CRY then suppress CLOCK‐BMAL1 transcriptional activity through a negative feedback mechanism, and this loop cycles once every 24 h to generate a circadian rhythm (Figure 1). The genes involved in this circuit are referred to as ‘core clock genes’.

Core clock genes such as *Bmal1* and *Clock* generate circadian rhythms by regulating a group of genes with E‐box sequences that provide a rhythm underlying cellular functions. For example, in pancreatic islet β‐cells, BMAL1 and CLOCK directly regulate a group of genes related to insulin secretion, generating a distinct circadian rhythm for insulin secretion^4^. In addition to these direct regulatory factors, another set of transcription factors, referred to as clock output genes, transmit the signals from core clock genes to downstream effector genes. Clock output genes include DBP, TEF, HLF, and E4BP4. We and others have recently conducted rigorous studies of their effects on metabolism (Table 1). Herein, we discuss the roles of these clock output genes, focusing on glucose metabolism.

**Table 1:** Tissue‐specific function of output clock genes

	Target	Methods	Effect	References
E4BP4	Intestinal epithelial cells (IEC)	IEC specific E4BP4 knockout mouse	Facilitate lipid absorption	[3]
	Liver	Adenoviral gene transfer	Inhibit gluconeogenesis	[16]
	Liver	Liver specific transgenic mouse, adenoviral gene transfer	Increase gluconeogenesis	[17]
	Pancreatic beta cells	Beta‐cell specific transgenic mouse	Inhibit insulin secretion	[8]
DBP	Adipocyte	3T3L1 adipocyte	Promote adipocyte differentiation	[6]
	Whole body	DBP, TEF, and HLF knockout mice	Deficiency in detoxification	[18]
			Early aging, epilepsy‐prone	

### The roles of DBP/E4BP4

DBP and E4BP4 are primarily regulated by a network that includes core clock genes. The promoter region of the *Dbp* gene has a BMAL1/CLOCK binding region, known as a cis‐regulatory element, referred to as E‐Box. The translated DBP binds to the DNA recognition site called D‐Box and thereby transmits signals to downstream genes. On the other hand, the E4BP4 promoter has an RRE region to which ROR and REV‐ERB bind, and translated E4BP4 is known to bind to the same D‐Box as DBP. Although DBP and E4BP4 are not considered to be core clock genes responsible for the circadian rhythm, they may play a role in fine‐tuning the core network because the circadian rhythm cycle length varies depending on the levels of DBP and E4BP4 expressions^5^.

As mentioned above, DBP and E4BP4 are regulated by BMAL1/CLOCK and ROR, respectively. Since BMAL1/CLOCK and ROR are reciprocally expressed, DBP and E4BP4 also have a reciprocal expression pattern. Therefore, as DBP is a transcriptional activator and E4BP4 is a transcriptional repressor, genes that are regulated by these two genes through the common D‐Box will have large expression amplitudes. Gene expressions regulated by other output clock genes are also expected to have a large amplitude.

Several reports have described the relationships between output clock genes and metabolic regulation, although not as comprehensively as for the core clock genes, and controversies regarding the results persist (Table 1).

DBP has been suggested to play a role in both the function and differentiation of adipocytes. DBP reportedly increases PPAR‐γ expression in adipocytes and is involved in improving insulin resistance^6^. It has also been reported that DBP expression is decreased in the omental and mesenteric adipose tissues of patients with diabetes^7^. The role of DBP in other metabolic organs is not well understood and requires additional study. Our investigations have clearly demonstrated the role of E4BP4 in pancreatic ß‐cell function. Transgenic overexpression of E4BP4 in pancreatic ß‐cells was shown to impair glucose‐stimulated insulin secretion, resulting in glucose intolerance^8^. Glucose‐responsive intracellular calcium was elevated and intracellular ATP/ADP ratios were dysregulated. In addition, expressions of *ins1* and other genes involved in insulin secretion were decreased, indicating that E4BP4 negatively regulates insulin secretion via multiple mechanisms^8^.

The liver is one of the major organs maintaining metabolic homeostasis in animals. We showed increased E4BP4 expression in the liver induced marked insulin resistance. Hepatic E4BP4 transgenic mice had decreased AKT phosphorylation and increased gluconeogenesis in the liver. Interestingly, in this hepatic E4BP4 transgenic mouse model, insulin‐stimulated AKT phosphorylation in muscle tissue was impaired. This muscle insulin resistance was shown to be associated with increased free fatty acid flux from the liver and decreased fatty acid utilization as an energy source. These findings suggest E4BP4 to be an important metabolic regulator in the liver that modulates both hepatic and muscle insulin sensitivity during the feeding–fasting cycle. We are in the early stages of revealing the organ‐specific metabolic control mechanisms governed by output system clock genes.

### Mechanisms regulating DBP and E4BP4 expressions

DBP and E4BP4 are widely recognized as being regulated by BMAL1/CLOCK and REVERB/ROR, as described in detail above. However, it is noteworthy that E4BP4 expression is reportedly up‐regulated by inflammatory stimuli independently of REVERB/ROR‐mediated regulatory mechanisms, such as those associated with macrophages^9^, ^10^, ^11^.

**Figure 2:**
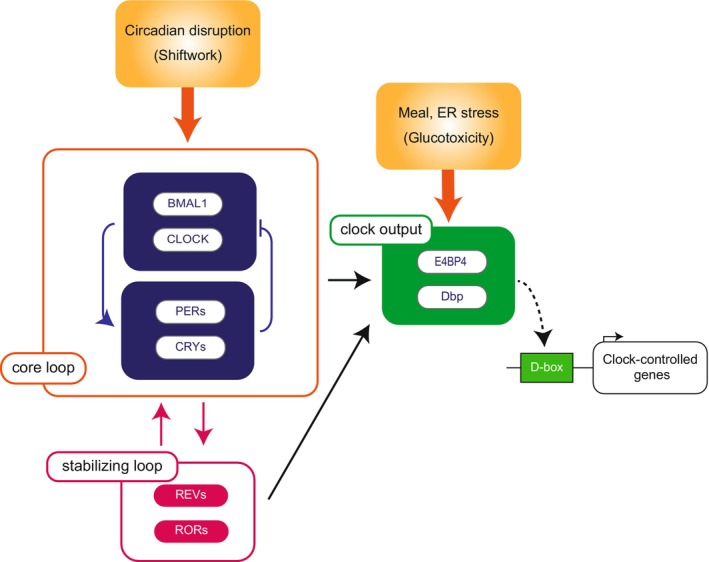
Schema of transcriptional regulation mechanism from core clock and stabilizing loops to clock‐controlled genes via clock output genes. Core loop signals regulate *Dbp* expression via E‐box elements, and stabilizing signals regulate E4BP4 expression via RRE elements. DBP and E4BP4 regulate downstream clock control genes by binding to the D‐box as transcriptional activators and repressors, respectively. Circadian disruption signals are transmitted to Coreloop, while meal and endoplasmic reticulum stress stimuli are thought to be transmitted to clock output genes.

Furthermore, we have observed *E4bp4* expression to be up‐regulated while *Dbp* expression is down‐regulated in islets of *Wfs1* knockout mice, a model of juvenile‐onset diabetes. Additionally, *E4bp4* was induced by endoplasmic reticulum stress, as its expression was also increased by treatment with tunicamycin and thapsigargin^8^. These findings provide insights into the regulation of output clock genes by factors other than core clock genes. This raises the possibility that in metabolic organs, entrainment by diet may affect primarily the output clock genes. Consequently, inputs such as light stimuli may impact the core clock genes, while information regarding diet, metabolism, and stress conditions may directly influence the output clock genes, *Dbp* and *E4bp4*. The possibility of direct input to the output clock genes *Dbp* and *E4bp4* has also been considered, and more detailed mechanistic insights are eagerly awaited (Figure 2).

### New approaches to nutritional therapy for patients with diabetes: Understanding circadian rhythms

In environments with artificial lighting, individuals frequently experience disruptions in their circadian rhythms due to irregularities in light exposure, sleep patterns, and eating habits. Research results suggest that such disruptions are associated with an increased risk for developing abnormal glucose metabolism^2^. Conversely, interventions targeting sleep have been shown to exert beneficial effects on glucose metabolism^12^.

Recent studies have underscored the impact of meal timing on circadian rhythms and physiological functions. Clock output genes, partially regulated by nutrient intake, might influence glucose metabolism via transcriptional regulatory mechanisms^13^. Differences in metabolic status have been documented, based on meal timing, with individuals who restrict calories within shorter time frames experiencing reduced risks of hepatic disease, obesity, and cardiometabolic disorders^14^.

Studies in mice suggest that time‐restricted feeding (RF) leads to prolonged fasting, which in turn activates various metabolic pathways in the liver, adipose tissue, and the intestines^13^, ^15^. While the precise mechanisms acting in human subjects remain unclear, RF has been shown to be associated with elevations in lipolysis and β‐oxidation, as well as alterations in the intestinal microflora composition.

Clock output genes such as *E4bp4* and *Dbp*, under dietary regulation, have the potential to serve as links between circadian rhythms and metabolic regulation. Nutritional therapy for diabetes has traditionally focused on caloric intake, but attention to meal timing, focusing particularly on time‐restricted diets, offers novel avenues for promising treatments.

## FUTURE PERSPECTIVES

Future research is anticipated to reveal the mechanisms underlying peripheral clock gene regulation, opening the way to the development of drugs targeting these genes without disrupting the central circadian rhythm. While drugs influencing core clock genes have shown metabolic benefits in mice, their use in patients with metabolic diseases requires further investigation aimed at minimizing any adverse effects on the central biological clock.

## DISCLOSURE

Yukio Tanizawa is an Editorial Board member of *Journal of Diabetes Investigation* and a co‐author of this article. To minimize bias, he was excluded from all editorial decision‐making related to the acceptance of this article for publication.
